# Supplementary material for: Real-time PCR detection of mixed Plasmodium ovale curtisi and wallikeri infections in human and mosquito hosts
Source: PLoS Negl Trop Dis. 2023 Dec 8;17(12):e0011274. doi: 10.1371/journal.pntd.0011274 (PMC10732364; doi:10.1371/journal.pntd.0011274)
Supplement: Attachment — Submitted filename: Response to Reviewers Letter.docx [file pntd.0011274.s002.docx]

September 1, 2023

PLOS Neglected Tropical Diseases

Dear Editor;

We would like to respond to the comments made on the submission of our manuscript titled “***Real-time PCR detection of mixed Plasmodium ovale curtisi and wallikeri species infections in human and mosquito hosts***”. Please note that where the manuscript has been changed, the text is provided in quotes, and the specific word changes are underlined.

**REVIEWER 1:**

1. **Comment:** “Title - species can be deleted; it is recommended to change to … *Plasmodium ovale curtisi* and *P. ovale wallikeri infections*”

**Response:**

This change has been made, and the line now reads:

“Real-time PCR detection of mixed *Plasmodium ovale curtisi* and *wallikeri* infections in human and mosquito hosts”

2. **Comment:** “Line 21 - malaria species is not correct. Malaria is a disease; pathogen/parasite/Plasmodium species cause diseases/infections.”

**Response:**

This change in the first line of the abstract has been made, and the line now reads:

“*Plasmodium ovale curtisi* (*Poc*) and ….represent distinct non-recombining *Plasmodium* species that are increasing in prevalence…”

3. **Comment:**“Methods - Please provide primer names and sequences (and bp sizes of PCR products)”

**Response:**

The primer names were previously listed in Table 1, and we did not list the sequences as they were already previously published. However, we have now listed the names and sequences under the relevant Methods sections:

**“**The nPCR assay published by Calderaro, et al [17] was performed as described using HotStart Taq (Qiagen). First round amplification of *Plasmodium* *ovale* inclusive of *Poc* and *Pow* used rPLU1 (TCAAAGATTAAGCCATGCAAGTGA) and rPLU5 (CCTGTTGTTGCCTTAAACTTC), resulting in a ~800 bp PCR product. Second round amplification for *Poc* or *Pow* species-specific detection was performed using rOVA1 (ATTTTGAAGAATACACTAGG) and rOVA2 for *P. ovale curtisi* (GGAAAAGGACACATTAATTGTATCCTAGTG), and rOVA1v (ATCTCTTTTGCTATTTTTTAGTATTGGAGA) and rOVA2v (GGAAAAGGACACTATAATGTATCCTAATA) for *P. ovale wallikeri*. This round resulted in a 787-789 and 782 bp product for *Poc* and *Pow*, respectively.

…To maximize detection sensitivity of both species, both assays were run in parallel to 50 cycles, instead of the originally published 45 and 55 cycles. For *P. ovale curtisi* amplification, OVA-F (TTTTGAAGAATACATTAGGATACAATTAATG) and OVA-R (CATCGTTCCTCTAAGAAGCTTTACAAT) were used along with OVA (VIC) probe (CCTTTTCCCTATTCTACTTAATTCGCAATTCATG). For *P. ovale wallikeri* amplification, OVA-Fv (TTTTGAAGAATATATTAGGATACATTATAG) and OVA-R were used along with Ovav (FAM) probe (CCTTTTCCCTTTTCTACTTAATTCGCTATTATG). Product sizes for *Poc* and *Pow* were 127 and 130 bp, respectively.”

4. **Comment:** “Methods/Discussion - Table 1 - There are several other protocols to discriminate PoC/PoW (e.g. Fuehrer et al. JCM; Lamien-Meda et al. Acta Tropica). Please check literature.”

**Response:**

The Fuehrer et al. 2012 article in JCM describes a useful assay to detect both *Poc* and *Pow* but does not focus on distinguishing the two species which is why it is not included as a reference in Table 1. We have revised text regarding Figure 1 to make this more clear.

“Previously published molecular assays for the detection and differentiation of *P. ovale* species were reviewed (**Table 1**).”

“Table 1. Published molecular assays to distinguish *P. ovale* species, *Po curtisi* (*Poc*) and *Po wallikeri* (*Pow*), and their application to clinical samples.”

Instead we have now referenced the Fuehrer JCM 2012 article in the second line of the Methods. The rOVA1/2 and rOVA1v/2v primers listed in Table 1 in the Fuehrer et al. 2014 review in JCM are the same ones first published by Calderaro et al. and are already listed in our Table 1. However, this is an important review that we have now also added as a reference in the second line of the Methods section:

“Based on initial testing, a nested PCR (nPCR) assay from Calderaro, et. al [[17,18]](https://paperpile.com/c/09Nqci/gkwC+t8sB) and real-time qPCR assays from Perandin, et al. [[19]](https://paperpile.com/c/09Nqci/xhUiO) and Calderaro, et. al [[20,21]](https://paperpile.com/c/09Nqci/UHEp+eg31), all targeting the small subunit RNA gene (18S rRNA), were selected for further assay development and comparison.”

18. [Fuehrer H-P, Stadler M-T, Buczolich K, Bloeschl I, Noedl H. Two techniques for simultaneous identification of Plasmodium ovale curtisi and Plasmodium ovale wallikeri by use of the small-subunit rRNA gene. J Clin Microbiol. 2012;50: 4100–4102.](http://paperpile.com/b/09Nqci/t8sB)

21.	[Fuehrer H-P, Noedl H. Recent advances in detection of Plasmodium ovale: implications of separation into the two species Plasmodium ovale wallikeri and Plasmodium ovale curtisi. J Clin Microbiol. 2014;52: 387–391.](http://paperpile.com/b/09Nqci/eg31)

The Lamien-Meda et al. article describes a high resolution melting assay for *P. ovale curtisi* and *P. ovale wallikeri* detection. It has been added to the list of real-time qPCR assays in Table 1.

**REVIEWER 2:**

1. **Comment:** “The authors have introduced an optimized protocol that effectively discriminates between Plasmodium ovale curtisi (Poc) and Plasmodium ovale wallikeri (Pow) in mixed P. ovale species infections. However, there are certain aspects regarding the description of the population (sampling) used for protocol optimization that require clarification. Notably, **the qualitative and quantitative details of the study population are not explicitly provided.** For instance, in the discussion of the modified PCR on lines 114 and 119, **the sample size remains unclear.**

**Response:**

We agree that we need to provide more details regarding the study population. We have now added text to the Methods section to describe the nature of the studies in Cameroon and Tanzania from which samples were drawn and relevant sample sizes:

“DNA extracted from blood samples from Cameroon (n=16) and Tanzania (n=22) previously identified as *P. ovale*-positive based on an 18S qPCR that detects both *P. ovale* species [[23]](https://paperpile.com/c/cmVUQB/Jaqw) were used to compare the performance of the selected nPCR and modified qPCR assays. The Cameroon study was a prospective hospital based cross-sectional survey in the three main health facilities in the Dschang Health District in the West region of Cameroon that enrolled 431 patients between June-September 2020 that underwent screening for malaria per the attending physician [[27]](https://paperpile.com/c/09Nqci/k1HH). Tanzanian samples were drawn from symptomatic children screened for a malaria therapeutic efficacy study at Yombo Clinic in Bagamoyo in 2016-2017 (YB- samples, [[28]](https://paperpile.com/c/09Nqci/vpsh)) as well as asymptomatic children and adults undergoing screening for a malaria transmission study from 2018-2019 [MqTZ- samples [[8,29]](https://paperpile.com/c/09Nqci/D8E7+GRN1Y)]. Samples with lower Po 18S Ct values were deliberately chosen for study.”

**Comment:** In order to enhance the methodology section, it is imperative to **present a more coherent and sequential flow of the protocol,** rather than a fragmented description of the modifications, accompanied by appropriate statistical treatment. Addressing these concerns will greatly enhance the transparency and reproducibility of the study.”

**Response:**

Text was added to provide more details about the protocol.

“To maximize detection sensitivity of both species, both assays were run in parallel to 50 cycles, instead of the originally published 45 and 55 cycles. For *P. ovale curtisi* amplification, OVA-F (TTTTGAAGAATACATTAGGATACAATTAATG) and OVA-R (CATCGTTCCTCTAAGAAGCTTTACAAT) were used along with OVA (VIC) probe (CCTTTTCCCTATTCTACTTAATTCGCAATTCATG). For *P. ovale wallikeri* amplification, OVA-Fv (TTTTGAAGAATATATTAGGATACATTATAG) and OVA-R were used along with Ovav (FAM) probe (CCTTTTCCCTTTTCTACTTAATTCGCTATTATG). A CFX Connect Real-Time PCR Detection System was used to perform the assay. A common annealing temperature of 52.8℃ was chosen for yielding similar Ct thresholds for detection of the same plasmid copy concentrations.”

2. **Comment:** “The **methodology for determining the sensitivity and specificity values presented in lines 183-184 requires further clarification**. It is not apparent how these values were derived, and a detailed explanation of true and false positives would enhance the transparency of the data provided. Additionally, it would be beneficial to **describe the criteria used for establishing the Ct value threshold.** Elaborating on these aspects will provide a clearer understanding of the methodology employed and strengthen the interpretation of the results.”

**Response:**

The way in which we defined the Ct value threshold for positivity was explained in lines 138-140:

“Since distilled water negative controls also sometimes demonstrated late cycle amplification for the Poc assay (mean Ct = 49.4, range 49.2-49.9), Ct thresholds for positivity were set at Ct <49 for both species.”

The data used for calculating specificity comes from Table 2. We reported the limit of detection of the *Poc* and *Pow* assays to be in the 10^-1^ and 10^0^ range, respectively (first paragraph of Results section). Thus, to calculate ***Poc* specificity**, we used data from all 55 *Poc* runs depicted in Table 2, from 10^5^ down to and including 10^-1^ concentrations. There were no false positive *Pow* calls among these runs (0/55) when following the algorithm depicted in Figure 1, thus leading to a specificity of 100%, where specificity is defined as (# of true negatives) / (# of true negatives + # of false positives). Because there are cross-reactive false positive *Pow* Cts at the highest *Poc* concentrations (10^5^ to 10^3^, rarely replicated in clinical infections), we designed the algorithm so that when *Poc* Ct is <39, the presence of *Pow* is only counted if Pow Ct is <44.

To calculate ***Pow* specificity,** we used data from 45 *Pow* runs depicted in Table 2 from 10^5^ down to and including 10^0^ concentrations, based on the limit of detection of 4.5 plasmid copies/μL for the *Pow* assay. No false positive *Poc* calls were made (0/45) when the classification algorithm was used, also leading to a specificity of 100%. The two positive *Poc* results at 10^1^ and 10^0^ would not be counted due to Ct values >49, and any positive *Poc* Ct values at a concentration of 10^3^ plasmid copies/uL or higher also would not be counted due to the *Pow* Ct value of <39 and the Poc Ct values >44.

Calculating ***Poc* sensitivity** relies on the data depicted in Table 3, in which mixed infections were simulated. Because the *Poc* sensitivity was 0.6 plasmid copies/μL, we included all runs of *Poc* down to and including 10^-1^ plasmid copies/μL, resulting in a total of 335 samples for which *Poc* DNA was present. Out of these 335 samples, 280 returned a positive result for *Poc* for a sensitivity of 84%.

The ***Pow* sensitivity** calculation included runs down to down to and including 10^-1^ plasmid copies/μL (excluding the 10^-1^ runs) due to its limit of detection of 4.5 plasmid copies/μL. This yields a total of 255 samples tested for which *Pow* DNA was present. Out of these samples, 239 returned a positive result for *Pow* for a sensitivity of 94%.

We have now added text to make these specificity and sensitivity calculations more clear:

“The resulting proposed classification system (**Figure 1**) does not indicate the relative abundance of the two species, but simply whether both are present. This approach maintains 100% specificity for both species based on the simulated mono-infections in Table 2 (0/55 and 0/45 false positives for *Poc* and *Pow*, respectively, as we did not include the 10^-1^ plasmid copies/μL runs for *Pow* given the limit of detection of 4.5 plasmid copies/μL for the *Pow* assay). Based on the mock mixture data in Table 3, the modified qPCR assays paired with the proposed classification system achieve 84% sensitivity (280/335) for *Poc* (down to 10^-1^ plasmid copies/μL) and 94% sensitivity (239/255) for *Pow* (down to 10^0^ plasmid copies/μL)*.*

**Comment:** Additionally, it would be beneficial to describe the **criteria used for establishing the Ct value threshold.** Elaborating on these aspects will provide a clearer understanding of the methodology employed and strengthen the interpretation of the results.”

**Response:**

Please see our explanation to the previous comment regarding the selection of Ct value thresholds. This was explained in the manuscript with a reference to the Table 2 data:

“We found this is more likely to happen if the majority species is abundant (Ct <39), leading to a cross-reactive false positive result for the other species that is indistinguishable from a small concentration of the minor species (Ct >44) (see data in **Table 2**). The resulting proposed classification system (**Figure 1**) does not indicate the relative abundance of the two species, but simply whether both are present. ”

**REVIEWER 3:**

1. **Comment:** “The methods should give more details on equipment. What real-time PCR machine was used?

**Response:**

This has been added to the manuscript:

“Real-time PCR was carried out using FastStart Universal Probe Master mix (ROX,Roche) and published primer concentrations on a Bio-Rad CFX Connect Real-Time PCR Detection System.”

2. **Comment:** “Regarding ‘...detected down to 10^0^ 18S plasmid copies/uL of their respective P. ovale species, or the equivalent of 1-2 parasites/uL assuming 5-8 copies of 18S rRNA per genome’: Why not just say ‘one’? (10^0^ = 1). So this is saying that 1 plasmid copy is equivalent to 1-2 parasites assuming 5-8 copies per genome. It does not quite make sense. Unless it was supposed to be 10^1^.

**Response:**

The reviewer is correct in pointing out a discrepancy. We were attempting to be conservative in our estimations of sensitivity. We have now used a single estimate of 6 copies of 18S RNA per genome and rephrased the sentence accordingly, with the correct calculations:

“...both modified real-time PCR assays consistently detected down to 10^0^ 18S plasmid copies/$\mu$L of their respective *P. ovale* species, or the equivalent of roughly 0.2 parasites/$\mu$L assuming 6 copies of 18S rRNA per genome.”

**Comment:** Ct < 49 is still a pretty high threshold for positivity. Melting or dissociation curve analysis would have helped to confirm that amplicons are of target sequences.

**Response:**

We agree that this is a high threshold and that melting or dissociation curve analysis could add specificity to the assay. Since the original published *Pow* qPCR detection assay was run to 55 cycles for its threshold of positivity, and the *Poc* qPCR detection assay was run to 45 cycles, we felt comfortable setting a uniform threshold of positivity for <49 cycles based on our data. Our goal was to minimally modify and streamline the existing qPCR assays to facilitate accurate detection of mixed infections.

**Comment:** Though I understand the algorithm conceptually, it seems to be based on somewhat arbitrary thresholds. Further I fear that is based on the cross-activity first observed at 10^3^ (or 1000) copies. As the next previous dilution tested where no cross-activity was observed was at 10^2^ (100) copies, the threshold of cross-reactivity is not clear. It could be 200 copies…

**Response:**

The reviewer is right, but the Ct values for these 10-fold dilutions are already pretty close. We would rather be conservative. At most, we feel the Ct value threshold might change by 1-2 cycles. We chose to display as much of the raw data as possible in order for readers to make their own conclusions.

**Comment:** The observed mixed infection in this study, especially of the surprising mosquito experiment results, should be confirmed with amplicon sequencing.

**Response:**

While we agree that amplicon sequencing might help validate the mosquito experiment results, this would not be a trivial undertaking given the challenges we had working with the mosquito midgut-extracted DNA. As is, we employed a nested strategy with first round *Plasmodium* genus primers prior to *Poc* and *Pow*-specific qPCR assays in order to obtain results for the majority of samples. Also, our group previously used 18S rRNA amplicon sequencing to differentiate *Poc* and *Pow* species from blood samples (Mitchell et al. JID 2021, last row of Table 1), and found the data challenging to analyze. One-third of the samples could not be speciated due to strict filtering cutoffs that were used to be able to make sense of the data.

In our limitations paragraph in the Discussion, we devote sentences to acknowledging that the results of mosquito experiments were not expected and explain why we did not pursue targeted sequencing. We have now also added an additional phrase emphasizing the need for further confirmation of our results:

“Our finding that co-infection by both species was more common in mosquitoes than in human blood samples was surprising and needs to be validated with further studies.”

“... though we showed excellent concordance of the real-time PCR assays with nested PCR and Sanger sequencing, we did not attempt to sequence our predominantly-mixed midgut samples to verify the presence of both species due to the paucity of DNA available and the bioinformatic challenge of detecting mixed *Poc*/*Pow* infections by targeted sequencing [[23](https://paperpile.com/c/cmVUQB/Jaqw)].”

3. **Comment:** “The last two sentences of the introduction are results and discussion and should not be part of the introduction. The introduction should simply give background and rationale for the study and state the objectives.

The beginning of the discussion should be part of the introduction.

If at the beginning of a sentence, genus names should not be abbreviated, even if previously defined.

I don’t think that Tables should be formatted like Table 1 with all these paragraphs within cells. And the entire methods section should not be in landscape.”

**Response:**

We have made changes to avoid the use of abbreviation for genus names at the start of sentences. With regards to the other comments, respectfully, these appear to be stylistic and formatting preferences that we will leave up to the Editors as to whether changes are required. The introduction to the Discussion was meant to frame aspects of the Discussion after having reported all the results.

4. **Comment:** “Though I can see considerable work gone into this study, I must recommend a major revision to clarify the significance of the study and to validate some of the confusing findings.

It would be hopeful if the paper would early on and in the abstract/summary say why it is important to identify mixed Po infections. It is not clear to me.

The introduction should have more background on **why it is important to identify mixed Poc and Pow infections**. Is there a **reason that this could contribute to disease or understanding of epidemiology somehow?**”

**Response:**

It is important to identify mixed *Poc* and *Pow* infections, because a major unanswered question is whether these two species differ in aspects of their biology, epidemiology, and/or clinical presentation. Various studies have attributed differences in relapse patterns or the presence of thrombocytopenia, for instance, in *Poc* vs *Pow.* If mixed infections are more common than we realize and go undetected, we will wrongly ascribe certain phenotypes to one species or another.

The frequency of detection of mixed infections in the same human or mosquito hosts also raises the question of how often they are co-transmitted and also reflect the many opportunities for recombination. The possible degree of introgression between these two species has not been well studied given the paucity of genome data and lack of an in vitro culture system for either species.

One would not ask why it is important to detected mixed *P. falciparum*/*P. ovale* infections or *P. ovale/P. malariae* infections to accurately understand the epidemiology of different Plasmodium species.

Parts of the manuscript that highlight the significance of detecting mixed *Poc/Pow* infections include:

Last sentence of Abstract:

“The described real-time PCR approach can be used to identify the natural occurrence of mixed *Poc/Pow* infections in human and mosquito hosts and reveals that such co-infections and co-transmission are likely more common than appreciated. “

Last two sentences in first paragraph of Introduction:

“There is some evidence that the two species differ in their latency period or relapse periodicity, as well as their presentation in travelers [[3,9–12]](https://paperpile.com/c/09Nqci/yb72A+TF68B+0g2E5+I4Unf+K6p2Q). Still, the extent to which *Poc* and *Pow* differ in their biology, epidemiology, and clinical manifestations in Africa, where they are most frequently found, remains an active area of research [[13,14]](https://paperpile.com/c/09Nqci/I9Y9z+w3pYy).”

Last sentences of Discussion:

“Recognition of the limitation of current assays for detecting co-infection and continued development of better, more facile diagnostics will improve our ability to understand whether and how these two species potentially differ in their epidemiology, biology, and clinical manifestations. Our findings suggest that the degree to which these closely related but sympatric species co-circulate within their human and mosquito hosts may be underappreciated.”

**EDITORIAL COMMENTS (found commented in the PDF):**

We have responded to the editorial comments made directly on the text of the manuscript in the order in which they appear.

1. “Could there be data on parasitemia in parasite/L? Especially with the possibility of cross reactivity at higher parasitemia?”

**Response:**

Parasitemia in parasites/L is not available for many of these previously published assays due to their qualitative nature. Nested PCR, for example, cannot quantify a parasitemia value. Where available, parasitemia range has been added.

2. “Align this section, assuming this is the Uganda data”

**Response:**

This change has been made in the updated manuscript.

3. “How many samples?”

**Response:**

The number of blood samples from which DNA was extracted that were previously identified as *P. ovale* positive was 38. We have added this to the Methods section:

“DNA extracted from blood samples from Cameroon (n=16) and Tanzania (n=22) previously identified as *P. ovale*-positive based on an 18S qPCR that detects both *P. ovale* species [[25]](https://paperpile.com/c/09Nqci/6TVJV) were used to compare the performance of the selected nPCR and modified qPCR assays.”

4. How many samples were used there?

**Response:**

We have added this number to the manuscript to the Methods section:

“Additionally, real-time PCR detection of *P. ovale* species was performed on DNA extracted from 17 *P. ovale*-positive mosquito midgut samples.”

5. How were these Ct cut-off values arrived at?

**Response:**

Please see our detailed response to Reviewer 2’s Comment #2 above.

In the context of this sentence, we use Ct<39 to describe a sample that is abundant for a particular species. In Table 2, 39 is the maximum Ct value at which we noticed cross-reactivity with false positive detection of the other species. We have more directly pointed readers to Table 2 to show this:

“Based on the performance of the qPCR assays in mono- and mixed infections, an algorithm was developed to prevent false-positive detection of a secondary species within a mixed *Poc/Pow* infection. We found that this is more likely to happen if the majority species is abundant (Ct <39), leading to a cross-reactive false positive result for the other species that is indistinguishable from a small concentration of the minor species (Ct >44) (see data in **Table 2**).

6. “How would you define sensitivity here? Is it in reference to specificity?”

**Response:**

We refer to sensitivity as the ability of an assay to avoid false negatives (the number of true positives detected by a particular assay / the number of total true positives). Specificity is the ability of an assay to avoid classifying false positives. This real-time PCR assay was able to avoid classifying any false positives, leading to its 100% specificity, which was the priority in its optimization. It was also able to mostly call true positives for both species, with 94% sensitivity for *P. ovale wallikeri*, compared to 84% sensitivity for *P. ovale curtisi*. For a more thorough explanation with how these values were arrived at, please see our detailed response to comment #2 by Reviewer 2.
