# Supplementary material for: Real-time PCR detection of mixed Plasmodium ovale curtisi and wallikeri infections in human and mosquito hosts
Source: PLoS Negl Trop Dis. 2023 Dec 8;17(12):e0011274. doi: 10.1371/journal.pntd.0011274 (PMC10732364; doi:10.1371/journal.pntd.0011274)
Supplement: Attachment — Submitted filename: Response to Reviewers Letter.docx [file pntd.0011274.s003.docx]

November 3, 2023

PLOS Neglected Tropical Diseases

Dear Editor;

We would like to respond to the comments made on the re-submission of our manuscript titled “***Real-time PCR detection of mixed Plasmodium ovale curtisi and wallikeri species infections in human and mosquito hosts***”. Please note that where the manuscript has been changed, the text is provided in quotes, and the specific word changes are underlined.

**REVIEWERS 1, 2, 3: No comments to address**

**EDITORIAL COMMENTS:**

We have responded to the comments left by the editorial team in the body of the email notifying us of the decision on the submitted manuscript.

1. “At the end of the introduction, the authors discusses results that have not yet been presented and conclusions appears to be ‘prematurely’ drawn.

‘Our optimized protocol for running parallel Poc and Pow species-specific qPCRs detected mixed Po species infection in a smaller proportion of blood samples from Tanzania and Cameroon. However, this percentage was much higher in Po-positive mosquito midgut samples derived from mosquito feeding studies in Tanzania, raising the possibility that mixed Poc/Pow infections may be more common than currently appreciated.’”

**Response:**

This portion of the introduction has been replaced with a brief overview of the implications of the findings from the study and their contributions to broader Po epidemiology instead of focusing on specific results and conclusions from the project. These sentences now read as follows:

“Since the existence of mixed *Plasmodium* infections of other species is well-documented, we suspected this was due to technical limitations rather than biological constraints. We adapted previously published nested and real-time PCR (qPCR) assays for *Po* species detection to establish criteria for defining when both *Poc* and *Pow* are present, and then applied these assays to *Po*-positive isolates from Africa. Our findings allow us to meaningfully contribute to the growing interest in precisely defining the prevalence of non-falciparum malaria species in sub-Saharan Africa by describing a refined real-time PCR technique able to distinguish *Poc* and *Pow* with a high degree of sensitivity and specificity.”

2. “In the discussion section, the information at the beginning fits better (strengthen the introduction) than with the discussion. Can the authors consider this suggestion?

‘Improved detection and rising prevalence of non-falciparum malaria species in sub-Saharan Africa [4-6,8] has spurred interest in better defining their epidemiology and biology [3,9,32,33]. Now recognized as two separate species, Plasmodium ovale curtisi (Poc) and Plasmodium ovale wallikeri (Pow) appear to circulate sympatrically, yet it appears <20 cases of *Poc*/*Pow* co-infection have been reported across >35 studies encompassing 1,515 *P. ovale* cases in the literature [3,13,15,16,22,26].’”

**Response:**

This portion at the start of the Discussion section has been moved and integrated into the introduction to provide a more specific background concerning the current state of the literature surrounding techniques for *Poc*/*Pow* differentiation. The Discussion now begins with a brief overview of the methods and results. The Introduction now reads as follows:

“Interestingly, though both *P. ovale* species circulate sympatrically in time and space, mixed infections of the two species have rarely been described, with <20 cases of *Poc*/*Pow* co-infection reported across >35 studies encompassing 1,515 *P. ovale* cases in the literature [[3,13,15,16]](https://paperpile.com/c/RRSCQy/QlO0C+igeiA+CogYV+ty4id). Since the existence of mixed *Plasmodium* infections of other species is well-documented, we suspected this was due to technical limitations rather than biological constraints. “
